# Readiness of Bystander Cardiopulmonary Resuscitation (BCPR) during the COVID-19 Pandemic: A Review

**DOI:** 10.3390/ijerph191710968

**Published:** 2022-09-02

**Authors:** Muhammad Fattah Fazel, Mohamad Haiqal Nizar Mohamad, Mohd Azmani Sahar, Norsham Juliana, Izuddin Fahmy Abu, Srijit Das

**Affiliations:** 1 Faculty of Medicine and Health Sciences, Universiti Sains Islam Malaysia, Nilai 71800, Malaysia; 2 Institute of Medical Science Technology, Universiti Kuala Lumpur, Kuala Lumpur 50250, Malaysia; 3 Department of Human and Clinical Anatomy, College of Medicine and Health Sciences, Sultan Qaboos University, Alkoudh, Muscat 123, Oman

**Keywords:** CPR, pandemic, bystander CPR, cardiac arrest, OHCA, COVID-19

## Abstract

Early cardiopulmonary resuscitation (CPR) is a strong link in the of survival for sudden cardiac arrest. Hence, bystander CPR (BPCR) plays an important role in curbing mortality and morbidity from out-of-hospital sudden cardiac arrest. However, the recent global Coronavirus disease 2019 (COVID-19) pandemic has impacted both public training and confidence in performing out-of-hospital CPR. This paper reviews detailed information from databases including Google Scholar, Scopus, PubMed and Web of Science on the readiness of BCPR during the pandemic. We also discussed the challenges bystanders encountered during the COVID-19 pandemic and the precautions to follow. Finally, we also highlighted the limitations which would benefit future endeavours in establishing well-planned and sustainable CPR training programs for the public. Therefore, regardless of the existing COVID-19 pandemic, BCPR must be emphasised to curb out-of-hospital cardiac arrest (OHCA) mortality.

## 1. Introduction

Coronavirus disease 2019 (COVID-19) was declared a pandemic by the World Health Organization (WHO) in March 2020 [1]. The event stormed the entire world, and its spread via the mode of aerosol transmission seriously impacted multiple clinical management of patients, including those requiring cardiopulmonary resuscitation (CPR) [2]. The uncertainty of the safest technique and anxiety of contracting the COVID-19 disease may cause bystander CPR (BPCR) to take a back seat. As described by Song et al., COVID-19 patients are also part of the susceptible groups that may develop sudden cardiac arrest. Therefore, in responding to the event of out-of-hospital cardiac arrest (OHCA), bystanders are exposed to the risk of infection [3]. Based on analyses in 2020, BCPR must be emphasised regardless, even in the midst of a pandemic; this benefits of this lifesaving endeavour outweigh the risks of COVID-19 infection [4].

The COVID-19 crisis brings out a new challenge in providing quality BCPR. It could be due to a lack of training provided due to the limitation of face-to-face gatherings. Meanwhile, it could be due to increased bystanders’ anxiety in performing the procedure due to the risk of infection [5]. As described by Pranata et al., the BCPR rate was reduced by almost half from its original count compared to the non-COVID-19 era in certain countries [6]. The situation worsens with the fear of possibly contracting the disease, resulting in a reluctance to perform CPR, contributing to higher OHCA mortality rates [7]. Reports have shown that COVID-19 contributes to an increased number of OHCA cases and directly affects the global survival rates [7]. Generally, the contributing factors towards the successfulness of a CPR include vigorous community CPR training, public knowledge, attitude and confidence towards the CPR, the availability of automated electronic defibrillators (AED) in common public spaces, and the ability to operate the AED machines [8].

Data prior to the global pandemic showed that in Malaysia alone, about 10 to 24 OHCA cases were reported each month [9]. Worldwide data shows that OHCA usually occurs at home (68.8%), where most cardiac arrests occur without any witnesses (57.1%), and only 39.3% of the population received BCPR [10]. Survival rates from OHCA in Malaysia were low at 1.6% compared to Korea at 9.8% [10]. The time taken for CPR to be administered also affects the survivability rates of patients. Early CPR administration was shown to be 33% effective in increasing survival rates as opposed to late CPR administration (20%) [11]. Based on all the data, it is imperative to maintain public confidence in performing quality BCPR. Standard operating procedures (SOPs) of social distancing and protective face masks will be our close companions for perhaps years ahead. Therefore, we gathered and reviewed the readiness for BCPR during the pandemic and its limitations to benefit future endeavours in establishing a well-planned and sustainable training program.

## 2. Materials and Methods

### 2.1. Source and Search Strategy

This review was designed and completed with ample, thorough reading and collection from Scopus, PubMed Web of Science databases OpenMD, and Science Direct. Additional studies were also identified by retrieval from the reference list of selected articles. Essential information has been summarised and included in our review and commentary.

### 2.2. Keyword Search

Keywords used for literature search include “Cardiac Pulmonary Resuscitation”, OR “CPR”, OR “Bystander CPR”, OR “Laypeople CPR” AND “Cardiac Arrest”, OR “Out of Hospital Cardiac Arrests” OR “OHCA” AND “Pandemic” OR “COVID-19”.

## 3. Readiness of Bystander CPR

### 3.1. Bystander CPR (BCPR) Training

Trainers use many approaches as educational means to train bystanders, such as using feedback devices, cardiopulmonary resuscitation (CPR)-sensing, high-fidelity simulation training, and refresher training programs [12,13]. Sadly, recent literature indicates that only 35% of trained bystanders performed CPR [14]. The unwillingness to perform CPR may be attributed to the diminishing skills of trainees as there are no requirements and emphasis for refresher CPR training courses among people in the community [15,16,17]. Moreover, many basic life support (BLS) classes only focus on compression-only CPR, not identifying signs of cardiac arrest [17]. In addition, demographic factors also play a role with regard to bystander CPR (BPCR). Here, studies showed that young and highly educated people are more likely to perform CPR [15], while family members are less prone to perform CPR [18]. Psychological factors also determined the willingness of bystanders to perform CPR, which include panic, general anxiety, wrong CPR techniques, legal consequences, and potentially harming the patients [19,20]. To counter these obstacles, public health services and many non-governmental organisations (NGO) are keen on providing and improving CPR training for communities, which were proven to be cost-effective and increased the willingness of bystanders to perform CPR [20]. Hence, CPR training must be considered essential, as data have shown that less training being imposed in a population correlates with low BCPR rates and low out-of-hospital cardiac arrest (OHCA) survival with decreased willingness to perform CPR [21].

### 3.2. Challenges during Pandemic

Survival rates of OHCA have seen an increasing trend worldwide in these past two decades [22]. However, new regulations in performing quality CPR during the pandemic era have been introduced to lower the risks of Coronavirus disease 2019 (COVID-19) transmission. The European Resuscitation Council has outlined a standard operating procedure (SOP) for CPR with a compression-only technique. If patients exhibit any potential COVID-19 symptoms, bystanders should only give defibrillation without chest compression, providing they only have droplet-precaution Personal Protective Equipment (PPE) [23]. It was estimated that the death rate for BCPR from COVID-19 is really low [4], and placing barriers, such as cloth, on patients’ mouths was suggested to lower the risk of COVID-19 transmission [24].

It was noted that performing CPR results in aerosol being dispersed in the air from patients through chest compression, hence posing risks of COVID-19 transmission to the bystanders even without mouth-to-mouth ventilation [8,22,25]. Although studies reported the increase of OHCA cases during the COVID-19 pandemic, [26,27,28] efforts in carrying out resuscitation are still considered low [28]. Stay-at-home and movement control orders (MCO) practised worldwide may contribute to the decrease in BCPR in certain populations as people stayed at home and avoided social interactions and gatherings completely [29,30]. Table 1 highlights the number of BCPRs before and during the COVID-19 pandemic in various countries.

## 4. Highlights on BCPR to Prevent COVID-19 Infection

Although evidence is limited, existing Coronavirus disease 2019 (COVID-19) guidelines advise exercising caution while conducting bystander cardiopulmonary resuscitation (BPCR). The bystanders need to be aware of and adhere to proper guidelines in performing CPR, especially during the COVID-19 pandemic. The fear of contagious and infectious COVID-19 aerosol during resuscitation may reduce the numbers of those willing to provide BCPR and is often exacerbated by misinformation, especially on social media [38,39]. These factors may impact bystanders’ actions and willingness to perform CPR on urgent medical conditions, such as cardiac arrest, without having proper Personal Protective Equipment (PPE). Furthermore, older bystanders with comorbidities are even more discouraged from administering or participating as lay rescuers [6]. A basic life support (BLS) guideline released by the American Heart Association (AHA) supported BCPR to omit rescue breath and only to perform a “hands-only approach”, i.e., high-quality chest compression and place an automated external defibrillator (AED) as soon as possible for cardiac arrest victims [4,24,40]. The recommendation is in accord with a meta-analysis by Yao et al. [41]. Compression-only CPR resulted in a similar survival rate to the standard CPR in cardiac arrest victims.

**Figure 1:**
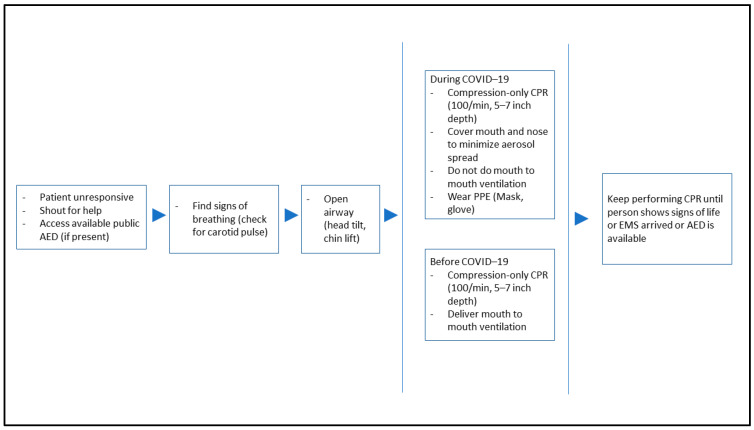
The differences between cardiopulmonary resuscitation (adult) standard operating procedures before and during the COVID-19 pandemic.

These guidance and instructions, however, are usually unknown to lay rescuers. The Resuscitation Council of the United Kingdom issued a recommendation on COVID-19 and resuscitation in the public community by urging rescuers to recognise cardiac arrest only by checking for the absence of signs of life and regular breathing, rather than using the look, listen, and sound method [38]. Another important recommendation for added protection when performing CPR on an unresponsive individual is that every effort should be made to cover the cardiac arrest individual’s face with a cloth to minimise the spread of high-risk aerosolising respiratory droplets and vice-versa for the rescuer [22,23,39]. Additional rescuers should avoid any close contact and stay within a safe distance of the victim’s airway and face until they are requested to perform CPR if the previous rescuer becomes fatigued [24,40]. These recommended precautions and early evidence on the possibility of COVID-19 transmission must be communicated to the public promptly to ensure the safety of rescuers while encouraging bystanders to save lives. Figure 1 displays the difference between cardiopulmonary resuscitation before and during COVID-19 pandemic. 

## 5. Discussion

The World Health Organization (WHO) announced the Coronavirus disease 2019 (COVID-19) outbreak, which emerged in Wuhan, China, to be the sixth public health emergency of international concern on 30 January 2020 [42,43]. Due to the outbreak, several countries began to announce movement control orders, home lockdown, social distancing, and isolation of quarantined infectious family members in order to dampen and lessen the COVID-19 contingency and R-naught [7,44].

The pandemic has affected the chances of survival of out-of-hospital cardiac arrest (OHCA) cases with regard to bystander cardiopulmonary resuscitation (BPCR). Prior to the COVID-19 pandemic, BCPR rates ranged from 40% to 55% worldwide [44], but the rate of BCPR was then halved when compared to the non-pandemic period [6]. According to the report, some countries or districts experienced an increase in BCPR during the COVID-19 pandemic compared to before the outbreak. This may be due to the lockdown and movement restrictions where OHCA cases are more likely to occur in residential settings. Additionally, the aid of BCPR was provided by the patient’s acquaintances, who were motivated to help regardless of fear of virus transmission [37]. The response and participation of BCPR are very important for the chain of survival as they double the chances of recovery from OHCA [45].

Several countries saw a decrease in BCPR due to the pandemic, e.g., France from 63.9% to 47.8% and Spain from 51.1% to 42.6% [26,28]. It has also been reported that the long emergency medical services (EMS) response time and the decrease in BCPR were associated with lower OHCA survival, possibly due to COVID-19 contagion and dissemination [40]. Based on a cohort investigation of OHCA attended by EMS in Washington, USA, from 1 January to 15 April 2020, during the pandemic, nearly 10% of cardiac arrest patients referred by EMS were COVID-19 positive [4,40]. Assuming a 10% transmission rate in the absence of Personal Protective Equipment (PPE), one rescuer may become infected after treating 100 patients [4].

Due to this concern, many countries have started to develop and evaluate the effectiveness of using a smartphone alerting system (SAS) for BCPR [46,47]. The alert system from SAS will help activate and trigger more trained BCPR volunteers who are qualified in basic life support (BLS) with prior knowledge in hygiene and understand how to handle infected patients safely while minimising and reducing the infection rate. Furthermore, by implementing this system, the chain of survival can be increased as the mass alert system will be able to find the first responder who serves as healthcare worker in emergency services or hospitals within the community in the event of OHCA with a faster response time [46,47,48].

A survey conducted by Mackler and colleagues investigated the willingness of paramedics to remain on care duty for a contagious disease, such as smallpox. The results revealed that only 4% of the respondents would remain on duty if there were no proper PPE and vaccine, while 39% would be prepared with proper PPE [49]. The nurses’ willingness to remain on care duty for COVID-19 patients was generally higher, as reported by Wu et al. [50]. The report revealed that of 128 nurses who declined to stay on care duty for COVID-19 patients, 26 (20.31%) declined due to the factor of infection probability and lack of PPE. In contrast, the remaining 92 (79.69%) declined due to age factors, pregnancy and maternity, fear of infection, unsupportive family and insufficient working skills [50]. Smallpox mortality was estimated to affect up to 300 million people in the 20th century [51,52] and the confirmed death by COVID-19 as of 7 April 2021, is 2.89 million, with 133 million cases of confirmed infection globally [53], which is certainly lower in comparison. However, it is assumed that proper and adequate PPE distributions will eventually increase the rate of BCPR response.

Even though several vaccines are currently in use and distributed worldwide, including Oxford-AstraZeneca, Pfizer-BioNTech, Sinovac, Sputnik V, Moderna, and Johnson & Johnson, the rate of infection and mortality is still increasing. This may be due to the emergence of new COVID-19 variants, such as B.1.1.7 (WHO labelled as Alpha), B.1.351 (WHO labelled as Beta), B.1.617.2 (WHO labelled as Delta), and P.1 (WHO labelled as Gamma) as reported by the Centers for Disease Control and Prevention (CDC) [54]. These variants are classified as Variants of Concern (VOC) by WHO, based on the evidence of increased transmissibility and severity of hospitalisations or death [54]. CDC also reported evidence on how these VOCs could interfere with diagnostics results and significantly decrease vaccines’ and treatments’ effectiveness. This may cause more unwillingness and decrease of BCPR in the event of OHCA, which may result in loss of more lives.

The risk of contracting the deadly COVID-19 viral disease generally contributes to the general public BCPR hesitancy to perform resuscitation on an unresponsive individual [40]. Previous goals to improve community BCPR rates are now jeopardised as the ‘new normal’ threatens to lower current global BCPR engagement and participants. There is no doubt that CPR is paramount in saving lives before further rescue by local medical authorities. The fact that it does save lives before and after the pandemic is still valid, but there must be a new standard operating procedure (SOP) to perform the protocol. The public community and BCPR must be continuously educated for them to be able to clearly consider the danger, risks, and best practices to approach in the event of OHCA during the COVID-19 pandemic. Perman suggested several considerations for BCPR or volunteers to perform CPR in current situations. It is important to prioritise the rescuer while adhering to every possible PPE in the event of OHCA, such as covering the face with a mask and considering eye protection whenever available [40]. To minimise and lessen the COVID-19 spread between rescuer and victim, the rescuer should always cover the patient’s mouth and nose using any mask or breathable clothing [40]. The rescuer should also only perform hands-only CPR, while other rescuers should stay within a safe distance of the victims’ airway until being signalled to approach or the need to perform the CPR [40]. The rescuer also should adhere to the proper way of decontamination after rescuing [40]. These suggestions also align with the American Heart Association’s (AHA) current guidance for CPR, which emphasises hands-on-only CPR [24].

Some limitations were encountered during this review. First, there was insufficient data on OHCA and BCPR between countries worldwide. Despite the fact that COVID-19 is considered a global disaster, each country’s pandemic severity varies. Therefore, published data on this issue during the pandemic was limited depending on each country’s policies and priorities. The data, as displayed in Table 1, were collected after an extensive search via several reliable search engines. The available data were also inconsistent as some came from the entire country, while others were from provinces or even parts of the country. Second, the data on BCPR were not collected from the same time period. Some types of research were conducted at the beginning of the pandemic, and other studiees were conducted at the highest point of the COVID-19 wave in their respective countries (e.g., the fourth wave of COVID-19 in India). Correspondingly, we cannot have confidence in drawing comparisons as researchers have conducted studies in different demographic settings and time periods, thus affecting their judgement during the research; andthey may have had different levels of knowledge about the disease.

## 6. Conclusions

Even though Coronavirus disease 2019 (COVID-19) has already passed its critical phase, it still can cause serious complications for susceptible individuals. Thus, bystander cardiopulmonary resuscitation (BPCR) is paramount in order to curb the problem of out-of-hospital cardiac arrest (OHCA). In addition, awareness programmes and community basic life support training are necessary for this post-pandemic era.

## Figures and Tables

**Table 1:** The number of bystander CPR (BCPR) before and during the COVID-19 pandemic as published in academic journals.

Region	BCPR before COVID-19 n (%)	BCPR during COVID-19 n (%)
Spain (Nationwide study) [28]	788 (51.1)	538 (42.6)
Sweden (Swedish registry) [31]	354 (66.2)	439 (74.8)
Singapore (Nationwide Study) [32]	240 (48.7)	278 (52.6)
Osaka, Japan [29]	356 (41.3)	272 (33)
Paris, France [26]	1165 (63.9)	239 (47.8)
USA		
(Detroit) [33]	73 (41)	117 (40)
(New York) [27]	441 (33)	1359 (34.1)
Victoria, Australia [34]	889 (73)	299 (78.7)
Italy		
(Province of Padua) [35]	15 (25)	10 (18)
(Lombardy) [36]	162 (35.7)	140 (24.5)
London, UK [37]	359 (52.6)	718 (63.3)
